# The Neural Correlates of Emotion Regulation by Implementation Intentions

**DOI:** 10.1371/journal.pone.0119500

**Published:** 2015-03-23

**Authors:** Glyn P. Hallam, Thomas L. Webb, Paschal Sheeran, Eleanor Miles, Iain D. Wilkinson, Michael D. Hunter, Anthony T. Barker, Peter W. R. Woodruff, Peter Totterdell, Kristen A. Lindquist, Tom F. D. Farrow

**Affiliations:** 1 SCANLab (Sheffield Cognition and Neuroimaging Laboratory), Academic Clinical Psychiatry, Department of Neuroscience, University of Sheffield, The Longley Centre, Northern General Hospital, Norwood Grange Drive, Sheffield, United Kingdom; 2 Department of Psychology, University of Sheffield, Western Bank, Sheffield, United Kingdom; 3 Department of Psychology, University of North Carolina, Chapel Hill, North Carolina, United States of America; 4 Academic Unit of Radiology, University of Sheffield, Royal Hallamshire Hospital, Glossop Road, Sheffield, United Kingdom; 5 Department of Medical Physics and Clinical Engineering, University of Sheffield, Royal Hallamshire Hospital, Glossop Road, Sheffield, United Kingdom; 6 Academic Clinical Neurology, Department of Neuroscience, University of Sheffield, Royal Hallamshire Hospital, Glossop Road, Sheffield, United Kingdom; 7 Department of Psychology, University of York, York, United Kingdom; 8 School of Psychology, University of Sussex, Pevensey Building, Falmer, United Kingdom; University of Gent, BELGIUM

## Abstract

Several studies have investigated the neural basis of effortful emotion regulation (ER) but the neural basis of automatic ER has been less comprehensively explored. The present study investigated the neural basis of automatic ER supported by ‘implementation intentions’. 40 healthy participants underwent fMRI while viewing emotion-eliciting images and used either a previously-taught effortful ER strategy, in the form of a goal intention (e.g., try to take a detached perspective), or a more automatic ER strategy, in the form of an implementation intention (e.g., “If I see something disgusting, then I will think these are just pixels on the screen!”), to regulate their emotional response. Whereas goal intention ER strategies were associated with activation of brain areas previously reported to be involved in effortful ER (including dorsolateral prefrontal cortex), ER strategies based on an implementation intention strategy were associated with activation of right inferior frontal gyrus and ventro-parietal cortex, which may reflect the attentional control processes automatically captured by the cue for action contained within the implementation intention. Goal intentions were also associated with less effective modulation of left amygdala, supporting the increased efficacy of ER under implementation intention instructions, which showed coupling of orbitofrontal cortex and amygdala. The findings support previous behavioural studies in suggesting that forming an implementation intention enables people to enact goal-directed responses with less effort and more efficiency.

## Introduction

Emotion regulation (ER) refers to the processes involved in the initiation, maintenance, and modification of the occurrence, intensity, and duration of feeling states [1–3]. ER is, for example, the process by which people overcome feelings of disgust in order to change a baby’s nappy, or overcome sadness to successfully deliver a eulogy. Regulating emotions in this deliberative, effortful manner can be effective in modulating emotional responses, but the effect of regulation on emotional outcomes is relatively modest [4] and may be associated with cognitive costs [5–6] and physiological resource depletion [7]. Thus, merely deciding to regulate emotions (i.e., forming a ‘goal intention’) is not necessarily sufficient to ensure effective and sustainable regulation [8].

One way to increase the likelihood that ER goals influence emotional outcomes is to form an implementation intention [9–10]. An implementation intention is an ‘if-then’ plan that links a specified cue (e.g., “If I see blood…”) with a *specific* goal-directed response (e.g., “…then I will look at the situation as if I was a doctor!”). Forming an implementation intention is effective because if-then plans (i) increase the accessibility of the cue specified in the if-part of the plan [11–14] and (ii) forge a strong link between the mental representation of the specified opportunity and the intended response [13–14]. Consequently, there is evidence that merely encountering the cue triggers the intended response in an immediate and efficient manner [13–15] without the need for conscious deliberation *in situ*. In short, an implementation intention recruits automatic processes in the service of a controlled strategy—a process known as 'strategic automaticity’ [16].

Schweiger Gallo and colleagues [17] (Study 1), showed that ER instructions in the form of an implementation intention (e.g., “If I see blood, then I will remain calm and relaxed!”) were more effective at modulating emotional outcomes than were ER instructions framed as a goal intention (“e.g., “I will not get disgusted!”). An EEG study [17] (Study 3) showed that the implementation intention influenced emotional responses early in the perceptual process (~100ms), which is consistent with the idea that implementation intentions reflect a relatively automatic process drawing on associations between a specified cue (e.g., “If I see blood…”) and a specific goal-directed response. There have now been approximately 30 studies investigating the effects of forming implementation intentions on emotional outcomes. A recent meta-analysis [8] indicated that forming implementation intentions is effective at modifying emotional outcomes, with a large effect relative to no-regulation instructions (*d*_+_ = 0.91) and a medium-sized effect relative to goal intention instructions (*d*_+_ = 0.53).

Despite evidence of the impact of forming implementation intentions on emotional outcomes, no studies to date have used fMRI to investigate the neural underpinnings of ER by implementation intentions. Understanding the neural basis of ER by implementation intentions is important both to understand the similarities and differences between ER by implementation intentions versus goal intentions, and to clarify whether if-then plans engender strategic automatization of ER. Previous research has reported that effortful ER strategies are associated with activation in prefrontal and temporal areas related to cognitive control including the dorsolateral prefrontal cortex (DLPFC), pre-supplementary motor area (pSMA), dorsal anterior cingulate cortex (ACC) and lateral temporal cortex [18–22]. Such effortful strategies decrease activity in subcortical limbic areas such as the amygdala (for meta-analyses, see [22–23]). Based on evidence that implementation intentions endow ER with features of automaticity (i.e., immediacy, efficiency, and redundancy of conscious intent; see [10] for a review), and theoretical proposals that automatic regulation of emotion might be supported by medial frontal areas such as orbitofrontal cortex (OFC) [24–26], ER by implementation intentions should show a different pattern of prefrontal recruitment. In particular, increased modulation of the amygdala and limbic system by ventromedial prefrontal cortex regions (such as the medial orbitofrontal cortex [mOFC]) should be observed.

The mOFC is well-suited to playing a role in automatic emotion regulation via implementation intentions, being a heteromodal association area that unites information from the sensory modalities, interoceptive information from the body, and representations of prior experiences [27] [28]. It is involved in associative processes such as classical conditioning and reversal learning [29], and processing contextually-relevant information [30]. The mOFC is also part of the brain’s “association system” [31–32] that is thought to use representations of prior experiences to make meaning of current sensations from the body and world [28] [33]. As such, it may contribute to the automatic regulation of emotion by helping a person to interpret visual sensations (an image) in light of learned associations (e.g., when I see the picture on screen, I will not react!), thereby reducing the intensity of emotional responding.

In the only previous study investigating the neural basis of implementation intentions using fMRI (but not involving emotion regulation), Gilbert and colleagues [34] compared prospective memory instructions that cued the intended response (e.g., “IF the same letter is on both sides, THEN I will press the middle button!”) with self-initiated instructions (goal intentions) that did not link the cue with a particular response (e.g., “IF the same letter is on both sides, THEN I can score 5 points!”). Although both sets of instructions were expressed in a contingent ‘if-then’ structure, Gilbert and colleagues proposed that only the cued instructions forged a direct link between the specified cue and the intended response in the manner associated with implementation intentions. As hypothesised by Gilbert and colleagues, performance on the prospective memory task was better under cued than under self-initiated instructions. Furthermore, performance under cued instructions was associated with increased activation within areas of the rostral prefrontal cortex including mPFC (BA10). Performance under self-initiated instructions, by comparison, was associated with greater activation of lateral BA10. Given that previous studies have associated medial BA 10 with environmentally-driven behaviour, and lateral BA 10 with tasks requiring attention to be diverted toward self-generated information [35–39] Gilbert and colleagues’ findings support the differing demands of cued performance versus self-initiated performance. Moreover, they suggest that the neural basis of ER under implementation intention versus goal intention may be distinct from one another.

### The Present Research

We used fMRI to investigate the neural basis of ER under implementation intentions versus goal intention instructions. Participants regulated their emotional responses to a series of disgust- or sadness-eliciting images. Prior to the fMRI scan, one-half of the participants were given a goal intention strategy to use, while the other half were given an ER strategy based on implementation intentions. We hypothesized that ER under implementation intentions would be more effective than under goal intentions as indexed by self reported affect. Neurally, we hypothesized that ER under goal intention instructions would recruit areas of the lateral prefrontal cortex, particularly DLPFC. In contrast, we hypothesized that ER under implementation intentions instructions would show a different pattern of prefrontal recruitment, relying more on medial frontal areas such as orbitofrontal cortex (OFC). We also hypothesized that the amygdala would show differential activation according to task instructions, given its role in the representation of emotional experiences and perceptions in general (for meta-analyses see [28] [40], but especially its decreased activity during successful emotion regulation (for meta-analyses see; [22–23]). Specifically we hypothesized that ER under implementation intentions would be associated with lower activity within the amygdala than ER under goal intentions. Finally, we hypothesized that connectivity between the amygdala and prefrontal cortex would vary as a function of implementation intentions versus goal intention instructions. Specifically, we hypothesized that the amygdala would show enhanced connectivity with regions such as DLPFC during ER under goal intentions, whereas ER supported by implementation intentions would show enhanced connectivity of the amygdala with more medial prefrontal regions, such as the OFC.

Given that emotionally laden images have previously been demonstrated to also impact peripheral physiological responses [41–42] and that voluntary ER to such stimuli has been shown to affect peripheral physiological responses such as the skin conductance response [43], we also collected skin conductance responses in order to investigate whether the nature of the instruction influenced autonomic responses. It was hypothesized that if ER under implementation intentions was associated with lower affect ratings, that this would also be reflected in concurrent reduced skin conductance responses.

## Methods

### Participants

Forty right-handed healthy participants (20 males; mean age = 20; range 18–23 yrs) were recruited from the local student population. Exclusion criteria were any current psychiatric or neurological disorder or contraindication to MR imaging. All participants spoke English as a first language and had normal or corrected-to-normal vision.

### Ethics Statement

Written informed consent was obtained from all participants and the study was approved by the University of Sheffield Research Ethics Committee.

### Pre-scan training

In the 48-hours prior to the scan, participants completed the Edinburgh Handedness Inventory [44] the Emotion Regulation Questionnaire (ERQ) [45] and the revised version of the Disgust Sensitivity questionnaire [46] (Table 1). These measures were included in order to ensure that participants in the goal intention and implementation intention groups were matched for handedness, trait emotional sensitivity and habitual emotion regulation style. Participants were randomised to either the goal intention or implementation intention group. A practice phase involved viewing three example stimuli to confirm that they understood the task.

**Table 1:** Baseline characteristics of participants (mean ± SD).

Mean (S.D)	Goal Intentions (N = 20)	Implementation Intentions (N = 20)	
Age in years	20 ±1	20 ±1	t = -.28, p = .95
Handedness	71 ±12	71 ±13	t = .19, p = .85
Disgust sensitivity^1^	36 ±16	36 ±15	t = .01, p = .99
ERQ reappraisal^2^	30 ±6	29 ±7	t = .41, p = .69
ERQ suppression^2^	19 ±5	20 ± 3	t = .78, p = .45

Participants in the goal intention and implementation intention groups were matched for age, handedness, baseline emotional sensitivity and habitual ER use

^1^Disgust Sensitivity questionnaire [46].

^2^Emotion Regulation questionnaire [45].

### Goal intention and implementation intention instructions

Both goal intention and implementation intention groups received two sets of instructions; each to be used in one functional run of the experiment. The first set of instructions told participants in the goal intention group that “When viewing pictures preceded by REAPPRAISE you should adopt a detached and unemotional attitude”. These instructions were based on reappraisal or distancing strategies widely used in previous studies of effortful ER [47–49]. Participants in the implementation intentions group received the further instruction: "If I see REAPPRAISE, then I will tell myself that ‘these are just pixels on a screen and the picture can't get to me!”. The second instruction told participants in the goal intention group that “Some pictures will be preceded by the word SUPPRESS. When viewing these pictures you should try to stop yourself from getting emotional. In other words, try to suppress any feelings you have when looking at the picture” (similar to the strategy used in [50]). Participants in the implementation intentions group received the further instruction: “If I see SUPPRESS, then ‘I will block out all bad feelings and just stay cool!'". Although the two sets of instructions for each condition were labelled as ‘reappraise’ or ‘suppress’, it should be noted that both might be interpreted as strategies traditionally defined as ‘distancing’ oneself from the stimulus in order to achieve emotional control. In this respect the ‘suppress’ instruction does not strictly correspond with the strategy of expressive suppression which involves avoiding any behavioural display of emotion [47]; suppression here is rather a form of cognitive suppression and relates to the experience rather than the expression of emotion [50]. Hence, the key difference between the ER instructions received by the two groups was that the implementation intentions instruction specified a cue (which could be quickly and accurately identified), the intended response (hence reducing the need for deliberation about how to respond) and tied the cue and response together in an ‘if-then’ format (thus promoting automatic response initiation).

### Stimuli

Images were chosen from the International Affective Picture System (IAPS) [51] on the basis of the normative arousal and valence ratings provided by [51], with the specificity of disgust or sadness selected on the basis of category-specific ratings from [52]. Disgust and sadness-eliciting images were included in order to not limit the instructions to a single emotion type. Nine neutral stimuli were also selected. Neutral stimuli were included primarily as a means of providing a buffer against the compound effects of repeated presentations of emotional stimuli. Details of the specific stimuli used are in S1 Dataset. The experiment was programmed in Presentation v14.4 (Neurobehavioural Systems, Inc) and stimuli were projected onto a screen that was viewable through a forward-mounted mirror above the MR scanner head coil.

### Paradigm details

**Fig 1:**
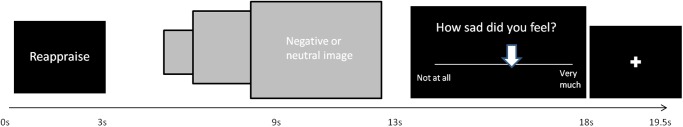
Events comprising each trial.

Each 19.5 second trial began with the presentation of the instruction to ‘attend, ‘reappraise’ or ‘suppress’ onscreen for 3 seconds. Participants were told that images preceded by the word ‘attend’ should be viewed naturally, allowing any feelings elicited by the image to occur (based on instructions used by [47–48]). For images preceded by the word ‘reappraise’ or ‘suppress’, participants were instructed to carry out the ER instructions that they had been taught. An image was then presented for 10 seconds, during the first 6-seconds of which the image appeared to advance towards the participant increasing from 80% of screen size to filling the screen. This manipulation was designed to increase the impact of the image and therefore the regulatory requirements [53]. Following each image, the question “How sad/disgusted did you feel while looking at the image?” appeared on screen for 5 seconds. Participants used the first two fingers of the right hand to press two buttons on a button box to move the cursor on the screen to the desired location on a Likert scale anchored by ‘not at all’ and ‘very much’. Finally, a fixation cross appeared for 1.5 seconds (Fig. 1).

Participants viewed a total of 45 stimuli in each run; 18 stimuli were designed to elicit disgust (9 viewed under instructions to regulate and 9 to attend), 18 stimuli were designed to elicit sadness (9 viewed under the instruction to regulate, and 9 to attend), and there were 9 neutral stimuli (attend only). Within each run, ‘pseudo-blocks’ of regulation type were created whereby participants followed the instruction to regulate or attend on between 2–4 successive trials, in order to increase the ease of using each strategy (i.e., reduce the demands of task switching). The order of stimuli was also pseudo-randomized such that no instruction type (reappraise, suppress or attend) appeared more than four times in succession, and no stimulus category (sad, disgust or neutral) appeared more than three times in succession.

Each image was only viewed once during each run. Different images were used for the two runs, such that participants viewed 90 images in total over the two runs; images in the two runs were matched for arousal and valence. The images used in each block, and whether an image was viewed under the instruction to regulate or attend, were counterbalanced across participants. The order of runs (reappraise and suppress) was also counter balanced within both groups (implementation intention and goal intention).

### fMRI data acquisition

During each functional run (lasting 14 minutes and 39 seconds), 293 volumes were obtained at 3T (Achieva, Philips Medical Systems, Best, NL) comprising 32 x 4mm thick contiguous slices (in-plane resolution 1.797x1.797mm) covering the entire cerebrum and cerebellum. A single-shot, gradient-recalled echo planar imaging (EPI) sequence was used: TR = 3 seconds; TE = 35 msecs; FOV = 240mm; in-plane matrix = 128x128mm). A high resolution T1-weighted structural scan was also collected for spatial normalisation (3D gradient echo, MP-RAGE, TR = 10.5ms; TE = 4.8ms; spatial resolution = 0.8mm^3^).

### fMRI data preprocessing

fMRI data were analyzed in SPM 8 (www.fil.ion.ac.uk/spm) implemented in MATLAB 7.1 (Mathworks Inc., Sherborn, MA). Images were motion-corrected, co-registered to each individual’s high-resolution T1-weighted scan, spatially normalised to the Montreal Neurological Institute (MNI) [54] single subject template using the unified segmentation approach [55], and smoothed with a Gaussian kernel (full-width half-maximum of 8mm). Of the 80 runs collected (40 subjects x 2 runs), 5 were excluded due to excessive head motion (>2mm). Blood-oxygen-level-dependant (BOLD) response was modelled to an event-related wave-form, convolved with a canonical haemodynamic response function and its temporal derivative. Individuals’ movement parameters were included as regressors in the contrast model to control for movement-related artefacts. Our contrast of interest focused on the 13-second period from when participants were first given the instruction to ‘regulate’ or ‘attend’ up to when the image was removed (Fig. 1). This 13-second window captured the entire regulation period, based on evidence that implementation intentions have rapid effects following identification of the relevant cue [17] and that these effects may last as long as the emotion-eliciting stimulus is present. At the level of the individual subject, epochs of ‘regulate’ were contrasted with epochs of ‘attend’. These first-level, fixed effects analyses were then taken forward to a second, group-level, flexible factorial design. Resulting contrasts from this flexible factorial design were examined at a both more conservative (p<0.05, FWE corrected) and more liberal (p<0.001 uncorrected) statistical thresholds. Finding objective and effective thresholds for voxelwise statistics derived from neuroimaging data has been a long-standing and ongoing issue of debate [56], though it has been suggested that optimal thresholds are lower than corrected for multiple comparison thresholds [57]. Specifically, we sought to fully investigate the accuracy of our *a priori* hypotheses while mitigating against over-reporting of Type 1 (false positive) results. While it has been reported [58] that brain-wide correction for multiple comparisons is unduly conservative for novel complex cognitive and affective social neuroscience processes such as were examined in the present study, we do not believe that use of such correction methods is an ‘all or nothing’ issue (i.e. that intermediate height and extent voxel-thresholds may be valuable in exploratory data analyses and suggesting future hypotheses). Hence, we have reported and interpreted activations at an uncorrected statistical threshold only if they were *a priori* hypothesised. We have additionally reported contrast values for relevant contrasts and utilised ROI analyses where appropriate. Co-ordinates for foci of activation were converted from MNI to Talairach by using the ‘mni2tal’ function within MATLAB.

#### Region of interest analysis

To examine the relationship between the activity within the amygdala and the efficacy of ER, a region of interest analysis was performed using the MarsBaR toolbox within SPM [59]. A mask of the left amygdala was created using the WFU PickAtlas toolbox [60], from which mean signal change during ER trials was extracted for each participant. This mean signal change was then correlated with participants’ self-reported reduction in affect during ER trials.

#### Connectivity analyses

For connectivity analyses, the time course of activation of amygdala from each individual was extracted when viewing all emotional images, (disgust and sad eliciting, but not neutral) regardless of regulation instruction. The search was constrained by a mask of the left amygdala (WFU Pickatlas toolbox within SPM8; [60]). The maximally activated voxel from this analysis was used as the centre of a 5mm radius sphere from which the first eigenvariate was extracted.

A psychophysiological interaction (PPI) term was produced by multiplying these amygdala time course vectors with the paradigm vector (regulate +1, attend-1, neutral 0). This PPI term was then re-entered as a regressor at the first level for each individual as an effect of interest, along with the time course and paradigm vectors as effects of no interest. This PPI term allowed examination of how the amygdala connectivity varied as a result of the instruction to regulate via goal intention or implementation intention strategies or attend. These first-level images were taken forward to a second-level, flexible factorial model with factors of subject and condition. From this second level contrast we investigated areas showing enhanced connectivity with the amygdala under the instruction of ‘regulation’ in comparison with ‘attend’. We also examined connectivity differences between goal intention and implementation intention ER-strategy processes. Results for the connectivity analyses are presented at p<.001 (uncorrected), with an extent threshold of 5 voxels.

### Skin Conductance Response (SCR)

MR-compatible SCR equipment was based on a battery-powered, electrically-isolated, same electrode configuration implementation of a previously published method [61]. SCRs were sampled at 20 Hz from the medial phalange of the left index and middle fingers, using 8mm diameter Ag / AgCl electrodes. SCR traces were analysed in Ledalab v.3.2.9 [62] using the Continuous Decomposition Analysis method to distinguish the phasic (driver) information from the underlying tonic sudomotor nerve activity. Raw SCR data were smoothed via convolution with a Hann window to reduce error noise and fitted to a bi-exponential Bateman function. Data were optimised by a conjugated gradient descent algorithm to reduce the error between them and the inbuilt SCR model. These processing steps allowed computation of a stimulus-locked ‘integrated skin conductance response’ (ISCR), a time-integration of the continuous phasic activity for each stimulus. This ISCR therefore represents an unbiased and time-sensitive measure of sympathetic activity in response to each stimulus [63]. For investigating whether implementation intention and goal intention ER strategies may be associated with different skin conductance response, ISCRs from participants in both groups were averaged across epochs, within-subject, using SPSS v19 (IBM Corp, Armonk, NY). Technical problems meant we were unable to obtain SCR recording from all participants; recording was obtained from a total of 26 participants (13 in each group).

## Results

### Self-reported affect

**Fig 2:**
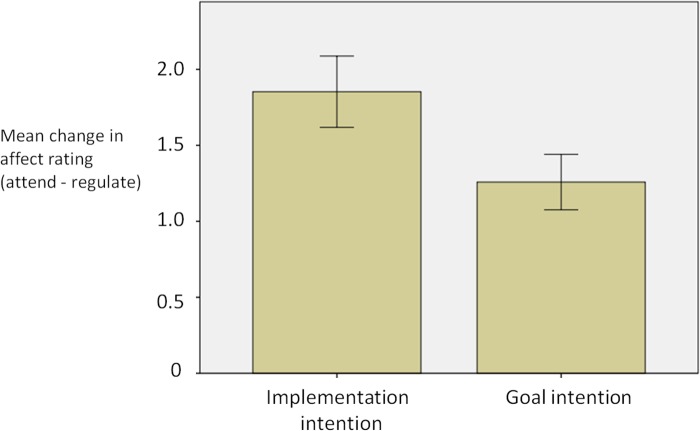
Mean change in affect ratings (and standard error) as a function of emotion regulation for the two groups. A greater mean change in affect rating is indicative of more successful emotion regulation (i.e. a larger reduction in affect ratings for the emotion regulation compared to not regulating emotion).

A 2 x 2 repeated measures ANOVA (within-subject factor of regulation condition [regulate or attend]; between-subject factor of instruction type [goal intention or implementation intention]) showed a main effect of regulation condition (‘regulate’ lower than ‘attend’; F(1,36) = 118.1, p<.0001). Implementation intention instructions were associated with larger changes in affect (attend-regulate) than goal intention instructions (mean change = 1.85 and 1.31 respectively; *t*(34) = 1.85, *p* < .05; Fig. 2), indicating that implementation intentions were more effective in reducing the intensity of emotional experience than were goal intentions. Both of the strategies were associated with lower affect ratings for the regulate condition compared to the attend condition (implementation intentions; *t*(19) = 7.28, p<.01; goal intentions; *t*(19) = 8.33, p<.01).

There was no difference between the reappraise and suppress strategy in reducing self-reported affect (*t*(37) = 1.36, p = .18). There was a significantly greater reduction in affect for the disgust images compared to the sad images (*t*(37) = 5.12, p<.05).

### Neuroimaging data

#### Conventional analyses. *ER under implementation intention instructions (ERii > attend)*

**Fig 3:**
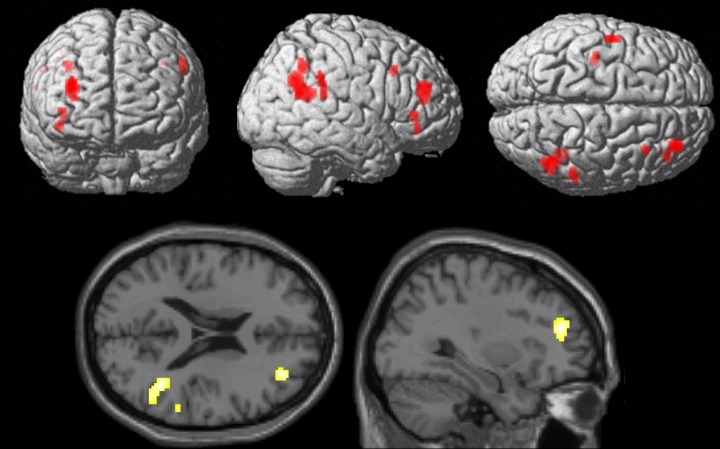
Brain areas more involved in emotion regulation by implementation intentions in comparison to attending to images. Data presented at p<.001 uncorrected, extent threshold 20 voxels.

ER under implementation intention instructions contrasted with the attend condition was associated with activations including right middle frontal gyrus (BA10; signal change = 0.43), right inferior frontal gyrus (BA47; signal change = 0.19), right superior temporal gyrus/temporo-parietal junction (BA39; signal change = 0.12), right inferior parietal lobule (BA40; signal change = 0.23), left precentral gyrus (BA6; signal change = 0.15) and left posterior cingulate gyrus (signal change = 0.12)(Table 2 and Fig. 3; p<0.001 uncorrected; extent threshold = 20 voxels).

**Table 2:** Areas activated in the contrast implementation intention > attend.

Area	Tal coordinates			Voxels	z value	Peak-level p (uncorr)
	X	Y	Z			
Rt. Middle frontal gyrus (BA10)	32	42	16	127	4.46	0.0001
Rt. Ventro-parietal cortex (BA39)	46	-57	30	327	4.40	0.0001
*Rt*. *Ventro-parietal cortex*	*40*	*-48*	*21*		*4*.*37*	0.0001
*Rt*. *Ventro-parietal cortex*	*48*	*-53*	*21*		*4*.*00*	0.0001
Lt. Pre-central gyrus (BA6)	-51	-6	37	54	4.15	0.0001
Lt. Post-central gyrus (BA2)	-36	-18	32	42	3.77	0.0001
Rt. Inferior parietal lobule (BA40)	53	-36	28	56	3.73	0.0001
Rt. Inferior frontal gyrus (BA47)	40	33	0	39	3.65	0.0001

Data presented at p<.001 (uncorrected) with an extent threshold of 20 voxels.

Co-ordinates are shown in standardized neuroanatomical space (Talairach and Tournoux, 1988 [64]). BA = Brodmann’s area. Lt. = left. Rt. = right. Post. = posterior. Co-ordinates in italics without a corresponding extent threshold refer to sub-clusters of the preceding activation

#### ER under goal intention instructions (ERgi > attend)

ER under goal intention instructions compared with the attend condition was associated with activation of areas including bilateral middle frontal gyrus (MFG; BA8/9; right MFG; signal change = 0.21, left MFG signal change = 0.30) and left superior temporal gyrus/temporo-parietal junction (BA39; signal change = 0.05) (Table 3, p<0.001 uncorrected; extent threshold = 20 voxels).

**Table 3:** Areas activated in the contrast goal intention > attend.

Area	Tal coordinates			Voxels	z value	Peak-level p (uncorr)
	X	Y	Z			
Rt. Middle frontal gyrus (BA8)	44	16	40	85	3.85	0.0001
Lt. Middle frontal gyrus (BA9)	-44	15	34	22	3.57	0.0001
Lt. Temporo-parietal junction (BA39)	-48	-57	25	37	3.44	0.0001
*Lt*. *Temporo-parietal junction (BA39)*	*-53*	*-51*	*23*		*3*.*35*	0.0001

Data presented at p<.001 (uncorrected) with an extent threshold of 20 voxels.

Co-ordinates are shown in standardized neuroanatomical space (Talairach and Tournoux, 1988 [64]). BA = Brodmann's area. Lt. = left. Rt. = right. post. = posterior. Co-ordinates in italics without a corresponding extent threshold refer to sub-clusters of the preceding activation

#### Direct comparison of ER under implementation intention with ER under goal intention

ER under implementation intention instructions (ERii > attend) compared with ER under goal intention instructions (ERgi > attend) was associated with activations including left precentral gyrus (BA6; signal change = 0.08), precuneus (signal change = 0.12), right superior temporal gyrus (BA39/22; signal change = 0.02), and right inferior parietal lobule (BA40; signal change = 0.13) (Table 4).

**Table 4:** Direct comparison of implementation intentions and goal intentions.

Area	Tal coordinates			Voxels	z value	Peak-level p (uncorr)
	X	Y	Z			
Implementation intention > goal intention						
Lt. Precentral gyrus (BA6)	-53	-8	35	105	3.66	0.0001
Lt. Precuneus (BA7)	-10	-52	52	44	3.58	0.0001
Rt. Superior temporal gyrus (BA39/22)	38	-48	21	35	3.22	0.001
Lt. Precentral gyrus (BA3)	-38	-20	34	41	3.09	0.001
Rt. Inferior parietal lobule (BA40)	56	-34	22	55	3.01	0.001
*Rt*. Inferior parietal lobule *(BA40)*	*50*	*-35*	*29*		*2*.*97*	0.001
*Rt*. Inferior parietal lobule *(BA40)*	*48*	*-37*	*42*		*2*.*65*	0.004
Goal intention > Implementation intention						
Lt. sgACC (BA25)	-10	19	-13	28	3.42	0.0001
Lt. Amygdala	-8	-5	-15	56	3.20	0.001
Lt. Superior frontal gyrus (BA8)	-10	30	48	34	3.01	0.001

Data presented at p<.001 (uncorrected) with an extent threshold of 20 voxels.

Co-ordinates are shown in standardized neuroanatomical space (Talairach and Tournoux, 1988 [64]). BA = Brodmann's area. Lt. = left. Rt. = right. sgACC. = subgenual anterior cingulate cortex. Co-ordinates in italics without a corresponding extent threshold refer to sub-clusters of the preceding activation

**Fig 4:**
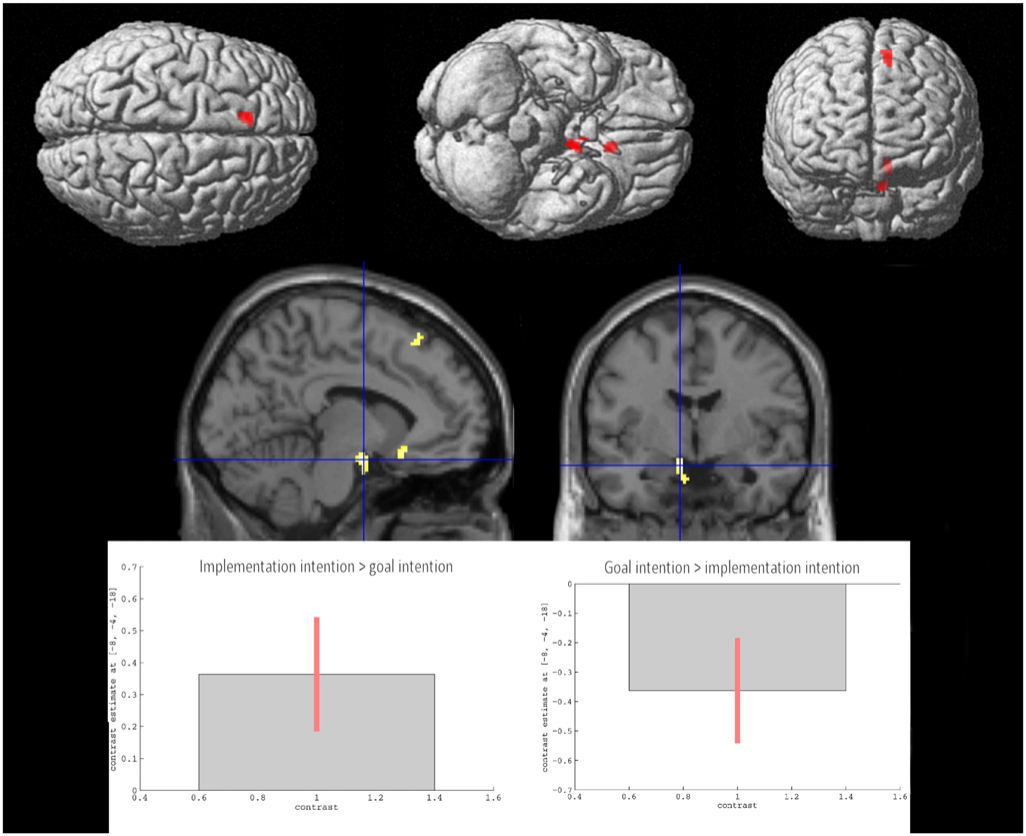
Brain areas showing greater activation under emotion regulation by goal intentions than implementation intentions (both strategies compared with attending to images). Data presented at p<.001 uncorrected, extent threshold 20 voxels.

ER under goal intention instructions compared with ER under implementation intention instructions was associated with greater activation of left sub-genual ACC (BA 25; signal change = 0.09), left superior frontal gyrus (BA8; signal change = 0.11), and the left amygdala (signal change = 0.11) (Fig. 4).

In order to further investigate the role of the left amygdala response we also conducted a region of interest analysis for the contrasts of attend > implementation intentions, and attend > goal intentions. This analysis revealed a greater left amygdala response to attending to the emotional images in comparison to emotion regulation for the implementation intentions group (t = 2.82, p<.01 corrected) but not for the goal intentions group (t = -0.52, p>.05 corrected), supporting the notion that ER by implementation intention was associated with greater down-regulation of left amygdala in comparison to ER under goal intention.

#### Correlation with behavioural data

**Fig 5:**
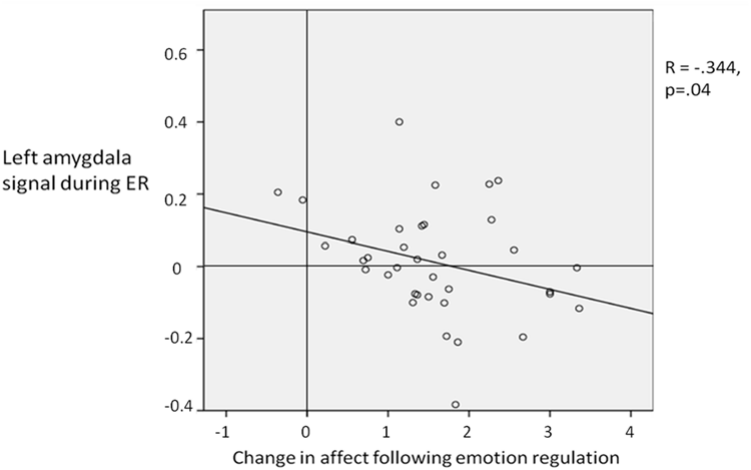
Correlation between the left amygdala signal during emotion regulation and the reduction in self report affect rating as a result of emotion regulation. The greater the reduction in affect rating rating is indicative of more successful emotion regulation (i.e. a larger reduction in affect ratings for the emotion regulation compared to not regulating emotion).

Lower self-reported affect during ER corresponded with relatively reduced left amygdala activity (ER > attend) (R =. -344; p = 0.04; Fig. 5). That is, more successful ER was associated with a greater reduction in left amygdala activity. When looking the two groups (implementation intentions and goal intention) it was found that this correlation was significant for the implementation intention group (R = -.512, p<.05) but not for the goal intentions group (R = -.316, p = .19), although a Fisher’s Z-transformation showed that the correlation coefficients for the two groups did not differ significantly (z = -0.56, two-tailed p = 0.58). There was no significant correlation found with the right amygdala for both groups together, or the implementation intentions and goal intentions group individually.

### Functional connectivity analyses

During ER under implementation intention instructions (ERii > attend), the left amygdala showed connectivity with right parahippocampal gyrus (BA38) and orbitofrontal cortex (BA11) (Table 5). During ER under goal intention instructions (ERgi > attend), the bilateral middle/superior frontal gyrus (BA8), right ACC (BA24), and left putamen showed functional connectivity with the left amygdala (Table 6).

**Table 5:** Areas demonstrating enhanced connectivity with the left amygdala under the condition of emotion regulation by implementation intention than under attend.

Area	Tal coordinates			Voxels	z value	Peak-level p (uncorr)
	X	Y	Z			
Rt. Parahippocampal gyrus (BA36)	32	-26	-24	30	3.4	0.001
Rt. Orbitofrontal cortex (BA11)	4	11	-21	9	3.12	0.001

Data presented at p<.001 (uncorrected) with an extent threshold of 5 voxels.

Co-ordinates are shown in standardized neuroanatomical space (Talairach and Tournoux, 1988 [64]). BA = Brodmann's area. Lt. = left. Rt. = right. post. = posterior.

**Table 6:** Areas demonstrating enhanced connectivity with the left amygdala under the condition of emotion regulation by goal intention than under attend.

Area	Tal coordinates			Voxels	z value	Peak-level p (uncorr)
	X	Y	Z			
Lt. Medial frontal gyrus (BA8)	-10	39	37	72	3.82	0.0001
Rt. Cingulate gyrus (BA32)	24	13	32	95	3.56	0.0001
*Rt*. *Cingulate gyrus (BA32)*	*25*	*19*	*40*		*2*.*90*	0.002
Lt. Anterior Cingulate (BA10)	-8	45	3	45	3.42	0.0001
Lt. Middle frontal gyrus (BA6)	-26	4	37	71	3.40	0.0001
Rt. Superior frontal gyrus (BA8)	17	32	50	9	3.04	0.001
Lt. Superior frontal gyrus (BA8)	-24	22	50	21	2.99	0.001
Lt. Putamen	-30	-15	10	25	2.94	0.002

Data presented at p<.001 (uncorrected) with an extent threshold of 5 voxels.

Co-ordinates are shown in standardized neuroanatomical space (Talairach and Tournoux, 1988 [64]). BA = Brodmann's area. Lt. = left. Rt. = right. post. = posterior. Co-ordinates in italics without a corresponding extent threshold refer to sub-clusters of the preceding activation

### Skin conductance response

A 2 x 3 repeated measures ANOVA revealed a main effect of regulation (regulate, attend, or neutral)(F(2,23) = 3.5, p<.05) but no interaction with instruction type (goal intention or implementation intention) (F(2,23) = 1.51, p = .24). A paired samples t-test showed that ISCR was significantly higher when attending to emotional images (mean ISCR = 1.318) compared to when attending to neutral images (mean = 1.06; t(25) = 2.33, p<.05).

## Discussion

We used fMRI to investigate the neural basis of two emotion regulation (ER) strategies that varied in their inherent automaticity. One half of the participants were instructed to use a general ‘goal intention’ strategy to regulate their emotions, while the other half of the participants used a more specific ‘implementation intention’ strategy. Behavioural and fMRI activation results supported our overarching hypothesis that implementation intentions are an effective means of achieving emotion regulation, which may in part be due to modulation of the amygdala by a relatively automatic process mediated by the orbitofrontal cortex.

Lower self-report affect among participants who formed an implementation intention compared to those who formed a goal intention supports previous studies suggesting that forming an implementation intention is an effective way to regulate affect [8][17]. The increased efficacy of the implementation intention was also supported by the neuroimaging data, which showed that activity in the amygdala was higher during ER under goal intention instructions than under implementation intention instructions (and that amygdala activity was higher in the contrast attend > implementation intention, but not for attend > goal intention). The general role of the amygdala in ER was further supported by a significant correlation between the reduction in amygdala signal during emotion regulation and the reduction in affect for the implementation intention group as well as the goal intention group [18] [65] [66] though we note that this analysis reveals that some participants showed an increase in amygdala activation whereas other participants showed a decrease. One possible explanation for this variability could be the fact that previous studies have shown that the amygdala tends to show an increase in response to the presentation of affective pictures [66–67] and that the signal may only begin to reduce after a delayed period during which the image is not presented. The current study maintained the image on screen throughout the entire regulation period, which may imply that the reduction in amygdala signal was not as great as for when the image is removed from view. It is also interesting within this study that only the left amygdala showed the differential reduction in activation as a function of emotion regulation instruction. Some other studies [18] [68] and one meta-analysis [23] have found similar modulation specifically within left amygdala during regulation of negative material. It is possible that this laterality effect may relate to the notion that the response of left amygdala to emotional stimuli is perhaps greater [69] or more sustained than that of the right [70–71]. It is also possible that the left amygdala may be more preferentially involved when the nature of the required processing for the emotional stimulus is more linguistic in nature [72]. However given that many other studies of goal intentions ER, including another recent meta-analysis [22], have also found an effect within the right as well as left amygdala, it is difficult to precisely account for this laterality effect. Future studies investigating emotion regulation by implementation intentions may be able to more systematically examine any role of the right amygdala by manipulating factors such as the length of presentation of the stimulus, or the linguistic complexity of the implementation intention instruction.

In line with previous studies of more effortful ER (e.g. [19] [20] [65]; for a meta-analysis, see [22]) we observed that goal intention instructions activated bilateral DLPFC and left temporoparietal junction (TPJ). These data are consistent with models of voluntary ER which suggest that it involves ‘executive control’ systems related to effortful processes. Indeed, recent findings support the idea that regions such as left DLPFC are involved in effortful executive processing generally, rather than being specific to ER [73–74]. In contrast, ER under implementation intention instructions showed a different pattern of prefrontal recruitment and was associated with activation in a network of right lateralised fronto-parietal regions. ER guided by an implementation intention is therefore dissociable from ER guided by goal intention to the extent that different neural systems are recruited.

Our hypothesis that amygdala-frontal connectivity would be evident during ER under goal intention instructions was confirmed and is consistent with a number of previous investigations of ER under goal intention instructions [18] [75]. Our data therefore provide further support for a model in which the efficacy of voluntary ER is driven by top-down modulation of the amygdala by frontal regions, particularly those involved in cognitive control. No such effect was found for ER under implementation intention instructions. Instead, we found connectivity during ER under implementation intention instructions between amygdala and perirhinal cortex/parahippocampal gyrus, and the orbitofrontal cortex (OFC). Connectivity between the left amygdala and the orbitofrontal cortex during ER under implementation intention instructions is noteworthy given the orbitofrontal cortex’s suggested role in automatic ER [24–26] [76]. Our data, therefore, suggest that some of the effects of ER supported by an implementation intention may be achieved through down-regulation of amygdala activity by the OFC. Other research has also suggested that connectivity between OFC and amygdala is associated with individual differences in ER [77] and that changes in this OFC-amygdala coupling may be a marker for risk of psychiatric diseases such as bipolar disorder [78], anxiety and depression [79].

It is noteworthy that activation of sub-genual anterior cingulate (sgACC; BA25) was found for the contrast of goal intention > implementation intention, given that this region has previously been associated with voluntary emotion regulation [18]. Activation in this region may therefore reflect the increased cognitive effort required for regulation under a goal intention, given that the appropriate action is not pre-planned in the same way as regulation under an implementation intention. This would support the proposed role of sgACC as a general mediator of ER in the absence of specific instructions towards a particular strategy [80].

Activation of the rIFG during ER under implementation intentions could reflect the involvement of the rIFG in the relevant inhibitory processes involved in emotion regulation such as inhibition of motor [81] and prepotent responses [82], emotional distraction [83], and the sustaining of attention [84]. However the concurrent increased activity within right ventro-parietal cortex (rvPC) results in a network that is strikingly similar to that observed during paradigms investigating attentional control [85]. Hampshire and colleagues report that the rIFG and right vPC are part of a network engaged by cues that trigger task-relevant behaviour. An interpretation of this finding, consistent with Hampshire and colleagues’ data, might therefore be that activation within such areas reflects a rapid orienting of attention that is captured by the relevant cue word appearing onscreen. This pattern of rIFG and rvPC activation was not seen during ER under a goal intention, supporting the idea that forming an implementation intention may heighten the accessibility of the cue word [12–14].

Taken together, our findings suggest that the automatic nature of ER under implementation intention instructions seems to be driven by the increased salience of the cue word (supported by rIFG), which allows efficient retrieval of the goal-directed response (supported by rvPC) that, in turn, facilitates more automatic regulation of the emotional response (in the amygdala) by systems known to be involved in more automatic ER (e.g., the OFC).

Activation within the left precentral gyrus during ER under implementation intention instructions was not one of our specific hypotheses. However it is possible that this activation reflects the involvement of the left precentral gyrus in the activation of verbal working memory [86–87]. Given that the right vPC (also activated during ER under implementation intention instructions) has also been implicated in retrieving previously learnt information for use in working memory [88], the neural substrates of ER under implementation intention instructions might therefore be interpreted in these terms to the extent that participants learn a particular piece of information (the if-then plan) which is then brought ‘online’ once the participant encounters the cue for action. Previous behavioural work has attempted to distinguish implementation intention from prospective memory instructions, and has suggested that implementation intention (but not prospective memory) instructions lead to automatic response initiation [89–91].

Although skin conductance response was higher for the emotional stimuli than neutral stimuli, further supporting the self-report data that the emotional stimuli did indeed induce emotional responses in participants, our data suggest that the nature of the ER strategy (goal- or implementation intention) did not modulate the autonomic response. It is possible that the relatively small difference in self-reported affect between the groups would not be reflected in significant SCR differences. Indeed, while we have previously shown a significant difference in ISCR between different categories of stimuli (threat vs. harm, [41]), we have not yet found differences between ER strategies applied to the same category of stimuli. It is also possible that the skin conductance response may have been influenced, particularly in the implementation intention group, by the salient cue. Indeed, the neural data indicates that the cue for action promoted attentional capture, which may also be reflected in the skin conductance response, which is known to exhibit an ‘orienting response’ to salient stimuli [92–93].

### Limitations

One limitation of the current study is that it used only young adult participants. Further studies using older populations are warranted given increasing evidence that the mechanisms of emotion regulation change in older populations [94] and that the nature of self-regulation more generally may be different in older populations [95]. Such studies could therefore examine whether the benefits of forming an implementation intention for regulating emotion are maintained throughout the lifespan.

We also acknowledge that we have utilised a relatively liberal height and extent threshold for our fMRI results, which may have led to reporting of some Type I (false positive) errors. The use of a mapwide false discovery rate (FDR) and family-wise error (FWE) of p<0.05 can be unduly conservative for novel complex cognitive and affective social neuroscience processes as were examined in this study [58]. We have therefore reported corrected results where possible and incorporated percent signal change values into our results. In light of reporting results from various analyses, we have exerted considerable caution to avoid over-interpretation of results, but hope that we have found a balance, such that the more tentative findings from this initial fMRI study of implementation intentions are reported so that they might feed into future hypotheses. Specifically, to address issues of power, a future study might consider using our specific imaging parameters (e.g. image matrix size, mask, voxel dimensions, and smoothing kernel) to conduct a (*post-hoc*) power analysis [96]. Finally, in the present study we have modelled the complex, and possibly temporally-variant process of ER as a single 13-second epoch, but acknowledge that in some regions of the brain, more transient responses may also be present. A future study might therefore consider whether an analysis modelling shorter duration events would reveal additional activations of interest. It is also worth noting that although the use of 19.5s trial length and TR = 3s constituted an ‘offset TR’ method of jittering, additional jittering of the length of the inter-trial interval (ITI) may have also led to a better characterisation of the shape of the hemodynamic response function [97–99].

### Conclusion

The present research demonstrates that ER supported by an implementation intention is associated with dissociable neural activations compared with ER under goal intention instructions. The effect of forming an implementation intention seems to be driven by processes involved in attentional control and may support more efficient regulation of areas such as the amygdala, by areas involved in automatic ER, such as OFC.

## Supporting Information

S1 Dataset Details of IAPS stimuli used. (DOCX)

## References

[1] Eisenberg N, Fabes RA, Guthrie IK, Reiser M. Dispositional emotionality and regulation: their role in predicting quality of social functioning. J Pers Soc Psychol. 2000;78: 136–157. 10653511 10.1037//0022-3514.78.1.136

[2] Gross JJ, Levenson RW. Emotional suppression: physiology, self-report, and expressive behavior. J Pers Soc Psychol. 1993;64: 970–986. 8326473 10.1037//0022-3514.64.6.970

[3] Gross JJ, Thompson RA. Emotion regulation: Conceptual foundations In: Gross JJ, editor. Handbook of Emotion Regulation. Guilford Press; 2007 pp. 3–24.

[4] Webb TL, Miles E, Sheeran P. Dealing with feeling: A meta-analysis of the effectiveness of strategies derived from the process model of emotion regulation. Psychol Bull. 2012;138: 775–808. 10.1037/a0027600 22582737

[5] Richards JM, Gross JJ. Composure at Any Cost? The Cognitive Consequences of Emotion Suppression. Personality and Social Psychology Bulletin. 1999;25: 1033–1044.

[6] Richards J, Gross JJ. Emotion regulation and memory: the cognitive costs of keeping one’s cool. J Pers Soc Psychol. 2000;79: 410–424. 10981843 10.1037//0022-3514.79.3.410

[7] Baumeister RF, Bratslavsky E, Muraven M, Tice DM. Ego depletion: is the active self a limited resource? J Pers Soc Psychol. 1998;74: 1252–1265. 9599441 10.1037//0022-3514.74.5.1252

[8] Webb TL, Schweiger Gallo I, Miles E, Gollwitzer PM, Sheeran P. Effective regulation of affect: An action control perspective on emotion regulation. Eur Rev Soc Psychol. 2012;23: 143–186.

[9] Gollwitzer PM. Implementation intentions: Strong effects of simple plans. American Psychologist. 1999;54: 493–503.

[10] Gollwitzer PM, Sheeran P. Advances in Experimental Social Psychology Volume 38 Adv Exp Soc Psychol. Elsevier; 2006;38: 69–119.

[11] Aarts H, Dijksterhuis A, Midden C. To plan or not to plan? Goal achievement or interrupting the performance of mundane behaviors. Eur J Soc Psychol. 1999;29: 971–979.

[12] Webb TL, Sheeran P. Identifying good opportunities to act: Implementation intentions and cue discrimination. Eur J Soc Psychol. 2004;34: 407–419.

[13] Webb TL, Sheeran P. How do implementation intentions promote goal attainment? A test of component processes. J Exp Soc Psychol. 2007;43: 295–302.

[14] Webb TL, Sheeran P. Mechanisms of implementation intention effects: the role of goal intentions, self-efficacy, and accessibility of plan components. Br J Soc Psychol. 2008;47: 373–95. 18096108 10.1348/014466607X267010

[15] Parks–Stamm EJ, Gollwitzer PM, Oettingen G. Action Control by Implementation Intentions: Effective Cue Detection and Efficient Response Initiation. Soc Cogn. 2007;25: 248–266.

[16] Gollwitzer PM, Schaal B. Metacognition in action: the importance of implementation intentions. Personal Soc Psychol Rev. 1998;2: 124–36. 10.1207/s15327957pspr0202_5 15647140

[17] Schweiger Gallo I, Keil A, McCulloch KC, Rockstroh B, Gollwitzer PM. Strategic automation of emotion regulation. J Pers Soc Psychol. 2009;96: 11–31. 10.1037/a0013460 19210061

[18] Banks SJ, Eddy KT, Angstadt M, Nathan PJ, Phan KL. Amygdala-frontal connectivity during emotion regulation. Soc Cogn Affect Neurosci. 2007;2: 303–312. 10.1093/scan/nsm029 18985136 PMC2566753

[19] Goldin PR, McRae K, Ramel W, Gross JJ. The neural bases of emotion regulation: reappraisal and suppression of negative emotion. Biol Psychiatry. 2008;63: 577–586. 17888411 10.1016/j.biopsych.2007.05.031 PMC2483789

[20] Kalisch R. The functional neuroanatomy of reappraisal: time matters. Neurosci Biobehav Rev. 2009;33: 1215–1226. 10.1016/j.neubiorev.2009.06.003 19539645

[21] McRae K, Hughes B, Chopra S, Gabrieli JDE, Gross JJ, Ochsner KN. The neural bases of distraction and reappraisal. J Cogn Neurosci. 2010;22: 248–262. 10.1162/jocn.2009.21243 19400679 PMC4136451

[22] Buhle JT, Silvers J a, Wager TD, Lopez R, Onyemekwu C, Kober H, et al Cognitive reappraisal of emotion: A meta-analysis of human neuroimaging studies. Cereb Cortex. 2013;24: 2981–2990. 10.1093/cercor/bht154 23765157 PMC4193464

[23] Diekhof EK, Geier K, Falkai P, Gruber O. Fear is only as deep as the mind allows: a coordinate-based meta-analysis of neuroimaging studies on the regulation of negative affect. Neuroimage. 2011;58: 275–285. 10.1016/j.neuroimage.2011.05.073 21669291

[24] Etkin A, Egner T, Kalisch R. Emotional processing in anterior cingulate and medial prefrontal cortex. Trends Cogn Sci. 2011;15: 85–93. 10.1016/j.tics.2010.11.004 21167765 PMC3035157

[25] Phillips ML, Ladouceur CD, Drevets WC. A neural model of voluntary and automatic emotion regulation: implications for understanding the pathophysiology and neurodevelopment of bipolar disorder. Mol Psychiatry. Nature Publishing Group; 2008;13: 833–857. 10.1038/mp.2008.65 PMC2745893 18574483

[26] Ochsner KN, Gross JJ. The cognitive control of emotion. Trends Cogn Sci. 2005;9: 242–249. 15866151 10.1016/j.tics.2005.03.010

[27] Barrett LF, Bar M. See it with feeling: affective predictions during object perception. Philos Trans R Soc B Biol Sci. 2009;364: 1325–1334. 10.1098/rstb.2008.0312 19528014 PMC2666711

[28] Lindquist KA, Wager TD, Kober H, Bliss-Moreau E, Barrett LF. The brain basis of emotion: a meta-analytic review. Behav Brain Sci. 2012;35: 121–143. 10.1017/S0140525X11000446 22617651 PMC4329228

[29] Hornak J, O’Doherty J, Bramham J, Rolls ET, Morris RG, Bullock PR, et al Reward-related reversal learning after surgical excisions in orbito-frontal or dorsolateral prefrontal cortex in humans. J Cogn Neurosci. 2004;16: 463–478. 15072681 10.1162/089892904322926791

[30] Shenhav A, Botvinick MM, Cohen JD. The expected value of control: an integrative theory of anterior cingulate cortex function. Neuron. Elsevier; 2013;79: 217–240. 10.1016/j.neuron.2013.07.007 PMC3767969 23889930

[31] Buckner RL, Andrews-Hanna JR, Schacter DL. The Brain’s Default Network. Ann N Y Acad Sci. 2008;1124: 1–38. 10.1196/annals.1440.011 18400922

[32] Shenhav A, Barrett LF, Bar M. Affective value and associative processing share a cortical substrate. Cogn Affect Behav Neurosci. 2013;13: 46–59. 10.3758/s13415-012-0128-4 23090717 PMC3557578

[33] Lindquist KA, Barrett LF. A functional architecture of the human brain: emerging insights from the science of emotion. Trends Cogn Sci. 2012;16: 533–540. 10.1016/j.tics.2012.09.005 23036719 PMC3482298

[34] Gilbert SJ, Gollwitzer PM, Cohen A-L, Burgess PW, Oettingen G. Separable brain systems supporting cued versus self-initiated realization of delayed intentions. J Exp Psychol Learn Mem Cogn. 2009;35: 905–915. 10.1037/a0015535 19586260

[35] Burgess PW, Scott SK, Frith CD. The role of the rostral frontal cortex (area 10) in prospective memory: a lateral versus medial dissociation. Neuropsychologia. 2003;41: 906–918. 12667527 10.1016/s0028-3932(02)00327-5

[36] Gilbert SJ, Frith CD, Burgess PW. Involvement of rostral prefrontal cortex in selection between stimulus-oriented and stimulus-independent thought. Eur J Neurosci. 2005;21: 1423–1431. 15813952 10.1111/j.1460-9568.2005.03981.x

[37] Gilbert SJ, Simons JS, Frith CD, Burgess PW. Performance-related activity in medial rostral prefrontal cortex (area 10) during low-demand tasks. J Exp Psychol Hum Percept Perform. 2006;32: 45–58. 16478325 10.1037/0096-1523.32.1.45

[38] Gilbert SJ, Spengler S, Simons JS, Frith CD, Burgess PW. Differential functions of lateral and medial rostral prefrontal cortex (area 10) revealed by brain-behavior associations. Cereb Cortex. 2006;16: 1783–1789. 16421331 10.1093/cercor/bhj113

[39] Gilbert SJ, Williamson IDM, Dumontheil I, Simons JS, Frith CD, Burgess PW. Distinct regions of medial rostral prefrontal cortex supporting social and nonsocial functions. Soc Cogn Affect Neurosci. 2007;2: 217–226. 10.1093/scan/nsm014 18985143 PMC2569804

[40] Kober H, Feldman Barrett L., Joseph J., Bliss-Moreau E., Lindquist K., Wager T.D. Functional grouping and cortical-subcortical interactions in emotion: A meta-analysis of neuroimaging studies. Neuroimage. 2008;42: 998–1031. 10.1016/j.neuroimage.2008.03.059 18579414 PMC2752702

[41] Farrow TFD, Johnson NK, Hunter MD, Barker AT, Wilkinson ID, Woodruff PWR. Neural correlates of the behavioral-autonomic interaction response to potentially threatening stimuli. Front Hum Neurosci. Frontiers; 2012;6: 349 10.3389/fnhum.2012.00349 23335893 PMC3546317

[42] Caseras X, Mataix-Cols D, An SK, Lawrence NS, Speckens A, Giampietro V, et al Sex Differences in Neural Responses to Disgusting Visual Stimuli: Implications for Disgust-Related Psychiatric Disorders. Biol Psychiatry. 2007;62: 464–471. 17306771 10.1016/j.biopsych.2006.10.030

[43] Driscoll D, Tranel D, Anderson SW. The effects of voluntary regulation of positive and negative emotion on psychophysiological responsiveness. Int J Psychophysiol. 2009;72: 61–66. 10.1016/j.ijpsycho.2008.03.012 18845192 PMC2676237

[44] Oldfield RC. The assessment and analysis of handedness: the Edinburgh inventory. Neuropsychologia. 1971;9: 97–113. 5146491 10.1016/0028-3932(71)90067-4

[45] Gross JJ, John OP. Individual differences in two emotion regulation processes: Implications for affect, relationships, and well-being. J Pers Soc Psychol. 2003;85: 348–362. 12916575 10.1037/0022-3514.85.2.348

[46] Olatunji BO, Williams NL, Tolin DF, Abramowitz JS, Sawchuk CN, Lohr JM, et al The Disgust Scale: item analysis, factor structure, and suggestions for refinement. Psychol Assess. 2007;19: 281–297. 17845120 10.1037/1040-3590.19.3.281

[47] Gross JJ. Antecedent-and response-focused emotion regulation: Divergent consequences for experience, expression, and physiology. J Pers Soc Psychol. 1998;74: 224–237. 9457784 10.1037//0022-3514.74.1.224

[48] Ochsner KN, Ray RD, Cooper JC, Robertson ER, Chopra S, Gabrieli JDE, et al For better or for worse: neural systems supporting the cognitive down- and up-regulation of negative emotion. Neuroimage. 2004;23: 483–499. 15488398 10.1016/j.neuroimage.2004.06.030

[49] Koenigsberg HW, Fan J, Ochsner KN, Liu X, Guise KG, Pizzarello S, et al Neural correlates of using distancing to regulate emotional responses to social situations. Neuropsychologia. 2010;48: 1813–1822. 10.1016/j.neuropsychologia.2010.03.002 20226799 PMC2905649

[50] Ohira H, Nomura M, Ichikawa N, Isowa T, Iidaka T, Sato A, et al Association of neural and physiological responses during voluntary emotion suppression. Neuroimage. 2006;29: 721–733. 16249100 10.1016/j.neuroimage.2005.08.047

[51] Lang PJ, Bradley MM, Cuthbert BN. International Affective Picture System (IAPS): Technical Manual and Affective Ratings. NIMH Center for the Study of Emotion and Attention. The Center for Research in Psychophysiology, University of Florida; 1997.

[52] Mikels JA, Fredrickson BL, Larkin GR, Lindberg CM, Maglio SJ, Reuter-Lorenz PA. Emotional category data on images from the International Affective Picture System. Behav Res Methods. 2005;37: 626–630. 16629294 10.3758/bf03192732 PMC1808555

[53] Mühlberger A, Neumann R, Wieser MJ, Pauli P. The impact of changes in spatial distance on emotional responses. Emotion. 2008;8: 192–198. 10.1037/1528-3542.8.2.192 18410193

[54] Mazziotta J, Toga A, Evans A, Fox P, Lancaster J, Zilles K, et al A four-dimensional probabilistic atlas of the human brain. J Am Med Informatics Assoc. 2001;8: 401–430. 11522763 10.1136/jamia.2001.0080401 PMC131040

[55] Ashburner J, Friston KJ. Unified segmentation. Neuroimage. 2005;26: 839–851. 15955494 10.1016/j.neuroimage.2005.02.018

[56] Genovese CR, Lazar NA, Nichols T. Thresholding of statistical maps in functional neuroimaging using the false discovery rate. Neuroimage. 2002;15: 870–878. 11906227 10.1006/nimg.2001.1037

[57] Thirion B, Pinel P, Mériaux S, Roche A, Dehaene S, Poline J-B. Analysis of a large fMRI cohort: Statistical and methodological issues for group analyses. Neuroimage. 2007;35: 105–120. 17239619 10.1016/j.neuroimage.2006.11.054

[58] Lieberman MD, Cunningham WA. Type I and Type II error concerns in fMRI research: re-balancing the scale. Soc Cogn Affect Neurosci. 2009;4: 423–428. 10.1093/scan/nsp052 20035017 PMC2799956

[59] Brett M, Anton JL, Valabregue R, Poline JB. Region of interest analysis using an SPM toolbox. Neuroimage. 2002;16: 497

[60] Maldjian JA, Laurienti PJ, Kraft RA, Burdette JH. An automated method for neuroanatomic and cytoarchitectonic atlas-based interrogation of fMRI data sets. Neuroimage. 2003;19: 1233–1239. 12880848 10.1016/s1053-8119(03)00169-1

[61] Shastri A, Lomarev MP, Nelson SJ, George MS, Holzwarth MR, Bohning DE. A low-cost system for monitoring skin conductance during functional MRI. J Magn Reson Imaging. 2001;14: 187–193. 11477679 10.1002/jmri.1171

[62] Benedek M, Kaernbach C. A continuous measure of phasic electrodermal activity. J Neurosci Methods. 2010;190: 80–91. 10.1016/j.jneumeth.2010.04.028 20451556 PMC2892750

[63] Benedek M, Kaernbach C. Decomposition of skin conductance data by means of nonnegative deconvolution. Psychophysiology. 2010;47: 647–658. 10.1111/j.1469-8986.2009.00972.x 20230512 PMC2904901

[64] Talairach J, Tournoux P. Co-planar stereotaxic atlas of the human brain. NY: Thieme Medical Publishers; 1988.

[65] Ochsner KN, Bunge SA, Gross JJ, Gabrieli JDE. Rethinking feelings: an fMRI study of the cognitive regulation of emotion. J Cogn Neurosci. 2002;14: 1215–1229. 12495527 10.1162/089892902760807212

[66] Schaefer SM, Jackson DC, Davidson RJ, Aguirre GK, Kimberg DY, Thompson-Schill SL. Modulation of amygdalar activity by the conscious regulation of negative emotion. J Cogn Neurosci. 2002;14: 913–921. 12191458 10.1162/089892902760191135

[67] Eippert F, Veit R, Weiskopf N, Erb M, Birbaumer N, Anders S. Regulation of emotional responses elicited by threat-related stimuli. Hum Brain Mapp. 2007;28: 409–423. 17133391 10.1002/hbm.20291 PMC6871321

[68] Phan KL, Fitzgerald DA, Nathan PJ, Moore GJ, Uhde TW, Tancer ME. Neural substrates for voluntary suppression of negative affect: a functional magnetic resonance imaging study. Biol Psychiatry. 2005;57: 210–219. 15691521 10.1016/j.biopsych.2004.10.030

[69] Baas D, Aleman A, Kahn RS. Lateralization of amygdala activation: a systematic review of functional neuroimaging studies. Brain Res Rev. 2004;45: 96–103. 15145620 10.1016/j.brainresrev.2004.02.004

[70] Gläscher J, Adolphs R. Processing of the arousal of subliminal and supraliminal emotional stimuli by the human amygdala. J Neurosci. 2003;23: 10274–10282. 14614086 10.1523/JNEUROSCI.23-32-10274.2003 PMC6741000

[71] Sergerie K, Chochol C, Armony JL. The role of the amygdala in emotional processing: a quantitative meta-analysis of functional neuroimaging studies. Neurosci Biobehav Rev. 2008;32: 811–830. 10.1016/j.neubiorev.2007.12.002 18316124

[72] Markowitsch HJ. Differential contribution of right and left amygdala to affective information processing. Behav Neurol. 1998;11: 233–244. 11568425 10.1155/1999/180434

[73] Golkar A, Lonsdorf TB, Olsson A, Lindstrom KM, Berrebi J, Fransson P, et al Distinct Contributions of the Dorsolateral Prefrontal and Orbitofrontal Cortex during Emotion Regulation. PLoS One. 2012;7: e48107 10.1371/journal.pone.0048107 23144849 PMC3492343

[74] Vanderhasselt M-A, De Raedt R, Baeken C, Leyman L, D’haenen H. The influence of rTMS over the left dorsolateral prefrontal cortex on Stroop task performance. Exp Brain Res. 2006;169: 279–282. 16418843 10.1007/s00221-005-0344-z

[75] Johnstone T, van Reekum CM, Urry HL, Kalin NH, Davidson RJ. Failure to regulate: counterproductive recruitment of top-down prefrontal-subcortical circuitry in major depression. J Neurosci. 2007;27: 8877–8884. 17699669 10.1523/JNEUROSCI.2063-07.2007 PMC6672169

[76] Etkin A, Wager TD. Functional Neuroimaging of Anxiety: A Meta-Analysis of Emotional Processing in PTSD, Social Anxiety Disorder, and Specific Phobia. Am J Psychiatry. 2007;164: 1476–1488. 17898336 10.1176/appi.ajp.2007.07030504 PMC3318959

[77] Fulwiler CE, King JA, Zhang N. Amygdala-orbitofrontal resting-state functional connectivity is associated with trait anger. Neuroreport. 2012;23: 606–610. 10.1097/WNR.0b013e3283551cfc 22617448 PMC4271793

[78] Versace A, Thompson WK, Zhou D, Almeida JRC, Hassel S, Klein CR, et al Abnormal left and right amygdala-orbitofrontal cortical functional connectivity to emotional faces: state versus trait vulnerability markers of depression in bipolar disorder. Biol Psychiatry. 2010;67: 422–31. 10.1016/j.biopsych.2009.11.025 20159144 PMC2835157

[79] Burghy CA, Stodola DE, Ruttle PL, Molloy EK, Armstrong JM, Oler JA, et al Developmental pathways to amygdala-prefrontal function and internalizing symptoms in adolescence. Nat Neurosci. 2012;15: 1736–1741. 10.1038/nn.3257 23143517 PMC3509229

[80] Nili U, Goldberg H, Weizman A, Dudai Y. Fear Thou Not: Activity of Frontal and Temporal Circuits in Moments of Real-Life Courage. Neuron. Elsevier Ltd; 2010;66: 949–962. 10.1016/j.neuron.2010.06.009 20620879

[81] Berkman ET, Burklund L, Lieberman MD. Inhibitory spillover: Intentional motor inhibition produces incidental limbic inhibition via right inferior frontal cortex. Neuroimage. 2009;47: 705–712. 10.1016/j.neuroimage.2009.04.084 19426813 PMC2700187

[82] Spence SA, Kaylor-Hughes C, Farrow TFD, Wilkinson ID. Speaking of secrets and lies: the contribution of ventrolateral prefrontal cortex to vocal deception. Neuroimage. 2008;40: 1411–1418. 10.1016/j.neuroimage.2008.01.035 18308586

[83] Dolcos F, Kragel P, Wang L, McCarthy G. Role of the inferior frontal cortex in coping with distracting emotions. Neuroreport. 2006;17: 1591–1594. 17001274 10.1097/01.wnr.0000236860.24081.be

[84] Hasenkamp W, Wilson-Mendenhall CD, Duncan E, Barsalou LW. Mind wandering and attention during focused meditation: a fine-grained temporal analysis of fluctuating cognitive states. Neuroimage. 2012;59: 750–760. 10.1016/j.neuroimage.2011.07.008 21782031

[85] Hampshire A, Chamberlain SR, Monti MM, Duncan J, Owen AM. The role of the right inferior frontal gyrus: inhibition and attentional control. Neuroimage. 2010;50: 1313–1319. 10.1016/j.neuroimage.2009.12.109 20056157 PMC2845804

[86] Henson RN, Burgess N, Frith CD. Recoding, storage, rehearsal and grouping in verbal short-term memory: an fMRI study. Neuropsychologia. 2000;38: 426–440. 10683393 10.1016/s0028-3932(99)00098-6

[87] Gruber O, von Cramon DY. The functional neuroanatomy of human working memory revisited. Evidence from 3-T fMRI studies using classical domain-specific interference tasks. Neuroimage. 2003;19: 797–809. 12880808 10.1016/s1053-8119(03)00089-2

[88] Cabeza R, Ciaramelli E, Moscovitch M. Cognitive contributions of the ventral parietal cortex: an integrative theoretical account. Trends Cogn Sci. 2012;16: 338–352. 10.1016/j.tics.2012.04.008 22609315 PMC3367024

[89] Rummel J, Einstein GO, Rampey H. Implementation-intention encoding in a prospective memory task enhances spontaneous retrieval of intentions. Memory. 2012;20: 803–17. 10.1080/09658211.2012.707214 22897132

[90] McDaniel MA, Howard DC, Butler KM. Implementation intentions facilitate prospective memory under high attention demands. Mem Cognit. 2008;36: 716–24. 18604955 10.3758/mc.36.4.716

[91] McDaniel MA, Scullin MK. Implementation intention encoding does not automatize prospective memory responding. Mem Cognit. 2010;38: 221–232. 10.3758/MC.38.2.221 20173194

[92] Frith C, Allen H. The skin conductance orienting response as an index of attention. Biol Psychol. 1983;17: 27–39. 6626635 10.1016/0301-0511(83)90064-9

[93] Williams LM, Brammer MJ, Skerrett D, Lagopolous J, Rennie C, Kozek K, et al The neural correlates of orienting: an integration of fMRI and skin conductance orienting. Neuroreport. 2000;11: 3011–3015. 11006985 10.1097/00001756-200009110-00037

[94] van Reekum CM, Schaefer SM, Lapate RC, Norris CJ, Greischar LL, Davidson RJ. Aging is associated with positive responding to neutral information but reduced recovery from negative information. Soc Cogn Affect Neurosci. 2011;6: 177–185. 10.1093/scan/nsq031 20385664 PMC3073385

[95] Dahm T, Neshat-Doost HT, Golden A-M, Horn E, Hagger M, Dalgleish T. Age shall not weary us: deleterious effects of self-regulation depletion are specific to younger adults Paterson K, editor. PLoS One. 2011;6: e26351 10.1371/journal.pone.0026351 22039469 PMC3200324

[96] Durnez J, Moerkerke B, Nichols TE. Post-hoc power estimation for topological inference in fMRI. Neuroimage. 2014;84: 45–64. 10.1016/j.neuroimage.2013.07.072 23927901

[97] Serences JT. A comparison of methods for characterizing the event-related BOLD timeseries in rapid fMRI. Neuroimage. 2004;21: 1690–700. 15050591 10.1016/j.neuroimage.2003.12.021

[98] Miezin FM, Maccotta L, Ollinger JM, Petersen SE, Buckner RL. Characterizing the hemodynamic response: effects of presentation rate, sampling procedure, and the possibility of ordering brain activity based on relative timing. Neuroimage. 2000;11: 735–759. 10860799 10.1006/nimg.2000.0568

[99] Dale AM. Optimal experimental design for event-related fMRI. Hum Brain Mapp. 1999;8: 109–14. 10524601 10.1002/(SICI)1097-0193(1999)8:2/3<109::AID-HBM7>3.0.CO;2-W PMC6873302

