# Supplementary material for: The Neural Correlates of Emotion Regulation by Implementation Intentions
Source: PLoS One. 2015 Mar 23;10(3):e0119500. doi: 10.1371/journal.pone.0119500 (PMC4370584; doi:10.1371/journal.pone.0119500)
Supplement: S1 Dataset — (DOCX) [file pone.0119500.s001.docx]

Disgust stimuli used were IAPS numbers: 2352.2, 2981, 3010, 3015, 3016, 3051, 3053, 3060, 3061, 3062, 3063, 3064, 3068, 3069, 3071, 3080, 3100, 3101, 3102, 3120, 3140, 3150, 3168, 3170, 3266, 3400, 7360, 8230, 9180, 9253, 9300, 9301, 9320, 9405, 9433, and 9570 (mean arousal = 6.25, mean valence = 2.02) . Sad stimuli were IAPS numbers: 2053, 2095, 2205, 2276, 2278, 2375.1, 2490, 2590, 2688, 2703, 2710, 2750, 2799, 2900, 3216, 3220, 3230, 3301, 3302, 3350, 6022, 6838, 7053, 9000, 9041, 9050, 9180, 9182, 9250, 9290, 9410, 9421, 9435, 9520, 9910, and 9921 (mean arousal = 5.29, mean valence = 2.55). Neutral stimuli were IAPS numbers: 2580, 2840, 5390, 5720, 7006, 7009, 7010, 7025, 7031, 7035, 7040, 7052, 7053, 7080, 7090, 7150, 7235, and 9008 (mean arousal = 2.71, mean valence = 5.01).
